# Fine-scale estimation of key life-history parameters of malaria vectors: implications for next-generation vector control technologies

**DOI:** 10.1186/s13071-021-04789-0

**Published:** 2021-06-08

**Authors:** Aaron L. Morris, Azra Ghani, Neil Ferguson

**Affiliations:** MRC Centre for Global Infectious Disease Analysis, School of Public Health, Imperial College London, Norfolk Place, London, W2 1PG UK

**Keywords:** Malaria, Mosquitos, Public health, Gene drive, Vector control, Population biology, Parameter estimation, Modelling

## Abstract

**Background:**

Mosquito control has the potential to significantly reduce malaria burden on a region, but to influence public health policy must also show cost-effectiveness. Gaps in our knowledge of mosquito population dynamics mean that mathematical modelling of vector control interventions have typically made simplifying assumptions about key aspects of mosquito ecology. Often, these assumptions can distort the predicted efficacy of vector control, particularly next-generation tools such as gene drive, which are highly sensitive to local mosquito dynamics.

**Methods:**

We developed a discrete-time stochastic mathematical model of mosquito population dynamics to explore the fine-scale behaviour of egg-laying and larval density dependence on parameter estimation. The model was fitted to longitudinal mosquito population count data using particle Markov chain Monte Carlo methods.

**Results:**

By modelling fine-scale behaviour of egg-laying under varying density dependence scenarios we refine our life history parameter estimates, and in particular we see how model assumptions affect population growth rate (*R*_m_), a crucial determinate of vector control efficacy.

**Conclusions:**

Subsequent application of these new parameter estimates to gene drive models show how the understanding and implementation of fine-scale processes, when deriving parameter estimates, may have a profound influence on successful vector control. The consequences of this may be of crucial interest when devising future public health policy.

**Graphic abstract:**

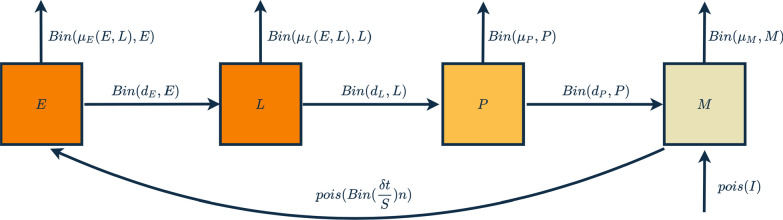

**Supplementary Information:**

The online version contains supplementary material available at 10.1186/s13071-021-04789-0.

## Background

Despite considerable progress, the global burden of disease caused by malaria remains high, and the demand for new, highly effective interventions is urgent [1]. To help reduce transmission, next-generation methods of control utilising genetic modification are being developed, which aim to reduce either the density or competence of vector populations [2]. With the release of genetically engineered organisms being strictly regulated, mathematical models remain a key resource for understanding their potential. However, gaps in our knowledge lead to an oversimplification of fine-scale mosquito dynamics, which can have a significant impact on our predictions of vector control efficacy.

The malaria parasite is transmitted principally by mosquitoes within the *Anopheles gambiae* complex, which comprises eight morphologically indistinguishable species [3]. In Africa, where 90% of malaria cases occur [1], *Anopheles gambiae *sensu stricto and *Anopheles funestus* are the dominant vectors in the majority of regions [4, 5], with *Anopheles arabiensis* and *Anopheles coluzzii* also being of significant importance [6]. Although each species exhibits some variation in behaviour and environmental tolerance [7–10], larval requirements for aquatic habitats mean that mosquito densities tend to peak at the times of year associated with high rainfall [11–13]. Interventions to reduce vector populations during these periods are an established method for reducing transmission—including larvicides [14], insecticide-impregnated bed nets [15] and pyrethrum spraying or trapping of adults [16]. These methods, however, have limitations, including the intensive use of chemicals, short half-lives of insecticides within the environment and the costs of continued redeployment. Next-generation “gene drive”-based interventions have the potential to overcome many of the problems inherent in traditional vector control [17]. They insert novel genes into a vector species which code for specific traits likely to cause either population suppression or reductions in vector competence, in a manner that allows those genes to propagate through the vector population. A promising technology in this field is the use of homing endonuclease genes (HEGs) [18], a class of nuclease genes found in simple single-celled organisms which can copy themselves from one chromosome to another. HEGs can be placed at specific sites on a chromosome and in a heterozygous individual produce an enzyme which cuts the DNA on the homolog of the HEG-bearing chromosome. When this site is repaired, the HEG-bearing chromosome is used as a template [19]. Such interventions have the potential to significantly reduce malaria transmission [2, 20–22], but are also susceptible to the emergence of resistance [23] or reinvasion of wild-type into depopulated landscapes [24].

Modelling shows that the spatial invasion dynamics of gene drive systems are highly sensitive to the fine-scale dynamics of mosquito populations [20]. Uncertainty around key aspects of these dynamics makes reliable predictions of the impact and spread of gene drive technologies challenging. In previous studies, the reproductive potential of a mosquito population (typically denoted as *R*_m_) has been found to be a key driver of the efficacy of gene drive constructs [20, 21, 24, 25]. *R*_m_ is the maximum rate at which a population can grow in the absence of density-dependent constraints (i.e. in the infinitesimal population density limit). However, since real populations are discrete and clumped, and subject to differing regimes of resource competition, the theoretical limit represented by *R*_m_ may never be attained. Understanding how a more realistic representation of the mosquito life cycle affects estimates of *R*_m_ is therefore important for refining assessments of the likely impact of gene drive technologies.

Although not exclusively, *R*_m_ in mosquito populations is often limited principally at the aquatic stages by density-dependent larval mortality, characterised by two parameters: the baseline density-independent mortality rate and the carrying capacity of the local environment for mosquito larvae. Carrying capacity describes the maximum number of mosquito larvae that can be sustained by the resources available within the environment, whilst density dependence affects the mortality (and developmental delays) caused by intraspecific competition for these resources [26, 27]. The two are intrinsically linked—for example, decreasing carrying capacity would lead to an increase in resource competition and hence density-dependent effects. How mathematical models approach larval density dependence is currently limited by the few experimental studies that exist, and analysis of experimental data has shown both linear and quadratic relationships between density and mortality [26–28]. Recent modelling studies have accounted for a lack of empirical data by implementing flexible density dependence functions in models to simulate a range of scenarios [29, 30].

Although a number of approaches have been used to simulate density dependence, most models tend to assume that in the low-density limit (e.g. one mated female entering an environment otherwise empty of mosquitoes), density-dependent effects will not apply, and the population will grow at its theoretical maximum (i.e. *R*_m_). However, this is likely to overestimate *R*_m_, as it is inconsistent with how a mosquito lays eggs in the natural environment, where habitat heterogeneity, local environmental conditions and behavioural traits will cause females to lay eggs in temporally and spatially clumped batches [31]. This behaviour has been studied in both *Aedes* and *Anopheles* mosquitoes, in laboratory conditions [32–34] and in the wild [35–37].

In this paper, using a discrete-time stochastic mathematical model of mosquito population dynamics, we aim to identify the effects of egg “clumping” on the estimation of *R*_m_. Additionally, as any conferred density effects from clumping are intrinsically linked with the functional form of density dependence, we explore multiple density dependence regimes. From this we aim to identify whether clumped egg-laying and specific forms of density dependence provide a better model fit to observed data, and the implications associated *R*_m_ estimates have on gene drive technologies.

## Methods

### Mosquito population model

**Fig. 1:**
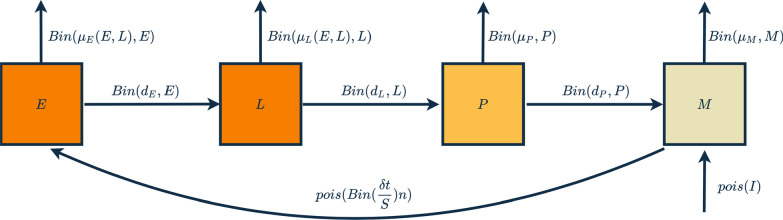
Model schematic showing transitions between stages, points of mortality and density-dependent mortality, immigration and egg-laying. Each arrow represents a Poisson draw, binomial or series of binomial events based on fitted probability parameters

We developed a discrete-time stochastic model that simulates the life cycle of a mosquito population. A schematic of the model is outlined in Fig. 1 and is described below.

The life cycle of a mosquito consists of four main stages: egg, larvae, pupae and adult. The first three stages are aquatic and, although there is variation in the wild, last approximately 5–14 days [38]. Larvae emerge from eggs and feed on algae, bacteria and other small particulate matter whilst developing through four separate larval instars, before metamorphosing into pupae and then finally adults. After maturation into the adult stage and having successfully mated, a female begins its first gonotrophic cycle. During the gonotrophic cycle, which lasts approximately 3 days, the mosquito takes a blood meal from a human or animal host, after which the blood is digested during a resting phase and used to develop the eggs in the ovaries. Once the eggs have matured, the mosquito is considered gravid and will search for a suitable site for oviposition. This process is then repeated until the eventual death of the adult.

As in previous models [28], to reduce complexity but maintain biological realism, we group the first two larval stages into a single class, early instar larval stage *E*, and the third and fourth stages into a late larval stage class *L.* Pupae *P* and adults *M* remain as single stages. Transition between early- and late-stage larvae and associated density-dependent mortality can be described as a binomial event occurring within discrete time $$\delta t$$. The probability of mortality or transition can be defined as $$\delta t$$ multiplied by the sum of the two respective rates, development to late-stage larvae ($$d_{E} )$$ and density-dependent mortality $$\mu_{E} \left( {E,L} \right)$$. A proportion $$\frac{{d_{E} }}{{\mu_{E} \left( {E,L} \right) + d_{E} }}$$ of all the larvae transitioning out of *E* are expected to enter the state *L*, again simulated by sampling from the corresponding binomial distribution. Similarly, transitions between late-stage larvae and pupae can be described with a probability $$d_{E}$$ and density-dependent mortality of $$\mu_{E} \left( {E,L} \right)$$. Pupae then develop into adults with a probability of $$d_{P}$$ and a density-independent probability of mortality $$\mu_{p}$$. Half of all emerging adults are assumed to be female, and male adults are not explicitly modelled (we assume that there are enough males to successfully mate with all females). For simplicity and model tractability we assume that adults die at an age-independent rate $$\mu_{{\text{m}}}$$ and have an approximate average life expectancy of 9–12 days in the wild [39]. Regular immigration of adult mosquitos from external populations is known to occur in most wild populations [40]; therefore, migration is modelled as a Poisson process with rate $$I$$. The input of new larvae into the system comes from eggs laid at a constant rate, $$M\beta \delta t$$, where $$\beta$$ is a fitted parameter for the average daily egg-laying rate of adult females.

The dynamics of a mosquito population can thus be described as a series of stochastic equations where ${\text{Bin}}\left( {p,n} \right)$ denotes a binomial draw with $p$ probability and $n$ trials, and ${\text{Poisson}}\left( \lambda \right)$ a draw from a Poisson distribution with given lambda.

$$E\left( {t + \delta t} \right) = E\left( t \right) - B_{E} + M\beta \delta t,$$

$$L\left( {t + \delta t} \right) = L\left( t \right) - B_{L} + {\text{Bin}}\left( {\frac{{d_{E} }}{{\mu_{E} \left( {E,L} \right) + d_{E} }},B_{E} } \right),$$

$$P\left( {t + \delta t} \right) = P\left( t \right) - B_{P} + {\text{Bin}}\left( {\frac{{d_{L} }}{{\mu_{L} \left( {E,L} \right) + d_{L} }},B_{L} } \right),$$

1 $$M\left( {t + \delta t} \right) = M\left( t \right) + \frac{1}{2}{\text{Bin}}\left( {\frac{{d_{P} }}{{d_{P} + \mu_{P} }},B_{P} } \right) - {\text{Bin}}\left( {\mu_{M} \delta t,M} \right) + {\text{Pois}}\left( { I} \right),$$

where

$$B_{E} = {\text{Bin}}\left( {\left( {d_{E} + \mu_{E} \left( {E,L} \right)} \right)\delta t,E} \right),$$

$$B_{L} = {\text{Bin}}\left( {\left( {d_{L} + \mu_{L} \left( {E,L} \right)} \right)\delta t,L} \right),$$

$$B_{P} = {\text{Bin}}\left( {(d_{P} + \mu_{P} )\delta t,P} \right).$$

### Density-dependent mortality

Density dependence in mosquito larval stages results from the competition for resources during the early and late instars; however, pupae are not pressured by resources, as they do not feed. For simplicity, although adults may have additional density constraints, it is assumed in this model that adults are not constrained by density effects [41].

Density dependence for early-stage larvae *E*(*t*) and late-stage larvae *L*(*t*) at time $t$ can therefore be described as $\mu_{E} \left( {E\left( t \right),L\left( t \right)} \right)$ and $\mu_{L} \left( {E\left( t \right),L\left( t \right)} \right)$.

To quantify the effect of egg-laying under different potential systems of density dependence, three functional forms were considered within the model:

A linear relationship between larval density and density-dependent mortality, as used by White et al*.* [28]:

2 $$\mu_{E} \left( {E\left( t \right),L\left( t \right)} \right) = \mu_{E}^{0} \left( {1 + \frac{E\left( t \right) + L\left( t \right)}{{K\left( t \right)}}} \right)^{ } ,$$

$$\mu_{L} \left( {E\left( t \right),L\left( t \right)} \right) = \mu_{L}^{0} \left( {1 + \gamma \frac{E\left( t \right) + L\left( t \right)}{{K\left( t \right)}}} \right).$$

Density-dependent mortality, which grows as some (fitted) power ($\Omega$) of larval density, resulting in $\Omega$ determining the intensity of density dependence, where $\Omega$ < 1 intensity grows at a decreasing rate, whilst $\Omega$ > 1 intensity grows at an increasing rate.

3 $$\mu_{E} \left( {E\left( t \right),L\left( t \right)} \right) = \mu_{E}^{0} \left( {1 + \frac{E\left( t \right) + L\left( t \right)}{{K\left( t \right)}}} \right)^{ \Omega } ,$$

$$\mu_{L} \left( {E\left( t \right),L\left( t \right)} \right) = \mu_{L}^{0} \left( {1 + \gamma \frac{E\left( t \right) + L\left( t \right)}{{K\left( t \right)}}} \right)^{\Omega } .$$

Exponential density dependence, where mortality increases exponentially with larval population density:

4 $$\mu_{E} \left( {E\left( t \right),L\left( t \right)} \right) = \mu_{E}^{0} e^{{\left( {\frac{E\left( t \right) + L\left( t \right)}{{K\left( t \right)}}} \right)}} ,$$

$$\mu_{L} \left( {E\left( t \right),L\left( t \right)} \right) = \mu_{L}^{0} e^{{\left( {\gamma \frac{E\left( t \right) + L\left( t \right)}{{K\left( t \right)}}} \right)}} .$$

Here, $\mu_{E}^{0}$ and $\mu_{L}^{0}$ are the death rates for early and late instars at low densities, $K\left( t \right)$ is the environmental carrying capacity at time $t$ and $\gamma$ is the difference in effect of density dependence on late-stage instars compared with early-stage instars. Larval carrying capacity (*K*) was assumed to be proportional to rainfall from the previous $\tau$ days weighted by an exponential distribution with mean $2\tau$, where ${\text{rain}}\left( t \right)$ in millimeters is the daily rainfall and $\lambda$ is a village-specific scaling factor as used by White et al*.* [28]:

5 $$K\left( t \right) = \lambda \frac{1}{{\tau \left( {1 - e^{{ - {\raise0.7ex\hbox{$t$} \!\mathord{\left/ {\vphantom {t \tau }}\right.\kern-\nulldelimiterspace} \!\lower0.7ex\hbox{$\tau $}}}} } \right)}}\mathop \int \limits_{0}^{t} e^{{ - \left( {t - t^{\prime}} \right)/\tau }} {\text{rain}}\left( {t^{\prime}} \right){\text{d}}t^{\prime}.$$

As there were inconsistent records of temperature in the Garki project for the study sites, we assumed a single fixed value. Similarly to White et al. [28], we used the mean air temperature of 24 °C to obtain an approximate mean value for the water temperature of 28 °C, and based our priors on this value.

### Clumped egg-laying

To simulate clumped egg-laying, the input of eggs into the system ($M\beta \delta t)$ in Eq. (1) is replaced by a stochastic “clumping” process. Here, the probability of each adult female laying eggs during any one time step $\delta t$ is $\frac{\delta t}{S}$, where $S$ is the duration of the gonotrophic cycle. The total number of females laying eggs, $E_{x}$, is thus sampled from a binomial distribution $E_{x} = {\text{Bin}}\left( {\frac{\delta t}{S},M} \right)$. The number of eggs laid in that time step is then sampled from a Poisson with mean $E_{x} n$, where $n$ is the mean clutch size ${\text{Pois}}\left( {E_{x} n} \right)$. As $n$, unlike $\beta$, explicitly considers the gonotrophic cycle $S$, the relationship between the two values is dependent on the average number of gonotrophic cycles within a mosquito's lifespan ($\mu_{{\text{m}}}$); therefore, approximately, $n = \frac{{ \beta \left( {e^{{S\mu_{{\text{m}}} }} - 1} \right)}}{{\mu_{{\text{m}}} }}.$

### Data

The model was fitted to rainfall and mosquito catch data obtained from the Garki project [39]. The project was a World Health Organization-funded study into the effects of malaria and vector interventions during the 1970s and presents one of the most detailed longitudinal data sets for combined mosquito and meteorological data. The data consist of environmental, demographic and mosquito catch data from 24 villages over an approximately 5-year period. Time series of *Anopheles gambiae* caught by pyrethrum spray in houses were extracted for eight villages and the data pooled to the village level. From these villages, two were from control areas where no interventions were carried out after the first rainy season, allowing the data to be split into two successive seasons, giving a total of ten population time series.

### Parameter estimation

We used an adaptive particle Markov chain Monte Carlo algorithm (pMCMC) to directly fit our stochastic model of mosquito dynamics to the data collected during the Garki project. pMCMC is a form of MCMC which uses a particle filter component to calculate a marginal likelihood for parameter acceptance/rejection within a Metropolis-Hastings algorithm [42, 43]. To account for the overdispersion and fractional sampling of the population, a beta-binomial likelihood function was used, with the parameter accounting for overdispersion $r$ and the fraction of the population sampled $p$ being fitted (see Additional file 1 for details of the pMCMC algorithm and likelihood methods).

The number of particles and chain length in a pMCMC has no set rules, and there is a trade-off between minimising the noise in the model and maintaining a realistic compute time. For each scenario of density dependence and egg-laying, we looked at the decrease in variation for set parameters when increasing the particle number and estimated the time to convergence in the chains. Therefore, the pMCMC chain was run with 150 particles for 2,000,000 iterations, with a 100,000-iteration burn-in. Attempts to increase the particle number and achieve convergence when fitting with an exponential density dependence were not successful, so full chains were not run. Priors were based on lab and field studies detailed in White et al. [28]. Approximate initial conditions for each of the villages were obtained by assuming deterministic equilibrium, allowing the value of each state variable to be derived from the value of a single estimate of the initial value of *E*, *L*, *P*, or *M* (see Additional file 1). This single parameter was denoted as *z* and estimated for each village. Most parameters were assumed to be common to all villages and were estimated by fitting the model to the data from all villages simultaneously, except village-/season-specific scaling factors $\lambda_{{\left( {1 \ldots 10} \right)}}$ and initial condition parameters $z_{{\left( {1 \ldots 10} \right)}}$, which were assumed to be village- and season-specific. Median values of the posterior distributions from the pMCMC chains for each parameter were then used to simulate the models for plotting, and 95% credible intervals were obtained by repeatedly sampling the joint posterior distribution.

### Mosquito population growth rate

*R*_m_ in the context of this paper is considered as the population growth rate in the low-density limit, as quantified by the number of adult female offspring produced per adult female. To calculate this, two values were derived: a raw value in the absence of density dependence and a second value which included the effects of density dependence. To obtain a raw analytical value we followed White et al*.* [28], where a female laying $n$ viable eggs during an oviposition cycle can expect to oviposit approximately $n\left( {e^{{ - S\mu_{{\text{m}}} }} + e^{{ - 2S\mu_{{\text{m}}} }} + \cdots } \right) = \frac{n}{{e^{{S\mu_{{\text{m}}} }} - 1}}$ eggs, where $e^{{ - S\mu_{{\text{m}}} }}$, $e^{{ - 2S\mu_{{\text{m}}} }}$, etc., is the proportion surviving one and two gonotrophic cycles, respectively. The fraction surviving to adulthood can then be defined respectively for clumped and non-clumped egg-laying as:

6 $$R_{{\text{m}}} = \frac{1}{2}\left( {\frac{n}{{e^{{S\mu_{{\text{m}}} }} - 1}}} \right)\left( {\frac{1}{{1 + \mu_{{\frac{E}{{d_{E} }}}}^{0} }}} \right)\left( {\frac{1}{{1 + \frac{{\mu_{L}^{0} }}{{d_{L} }}}}} \right)\left( {\frac{1}{{1 + \frac{\mu P}{{d_{L} }}}}} \right),$$

7 $$R_{{\text{m}}} = \frac{1}{2}\left( {\frac{ \beta }{{\mu_{{\text{m}}} }}} \right)\left( {\frac{1}{{1 + \mu_{{\frac{E}{{d_{E} }}}}^{0} }}} \right)\left( {\frac{1}{{1 + \frac{{\mu_{L}^{0} }}{{d_{L} }}}}} \right)\left( {\frac{1}{{1 + \frac{\mu P}{{d_{L} }}}}} \right).$$

However, clumped egg-laying means larval populations always experience some density-dependent competition; thus the raw value for *R*_m_ never predicts actual population growth. Accounting for minimal density dependence is analytically challenging, however. We therefore evaluated *R*_m_ numerically by simulating the model for a single female mosquito until its death in a system empty of other adults and recorded the number of female offspring surviving until adulthood. Simulations were repeated using multiple random samples of parameter sets sampled from the pMCMC posterior to estimate credible intervals. To account for stochastic variation, the mean was taken from 50 simulations of each parameter set.

### Effect on gene drive

To illustrate the effect on vector control, we looked at a test case of the impact on gene drive from changes in both density dependence and the addition of clumped egg-laying, by reconstructing the gene drive model formulated by Deredec et al. [20]. This model considers HEGs, a class of selfish genetic elements which are one of the most credible gene drive methods currently being developed. HEG efficacy is determined by the number of HEGs being used and their homing rate (the fraction of potential recipient heterozygous chromosomes that require the HEG). We ran the model using parameter estimates from our results for each of our scenarios and established the impact on the required HEGs and homing rate for elimination.

## Results

### Density-dependent mortality

**Fig. 2:**
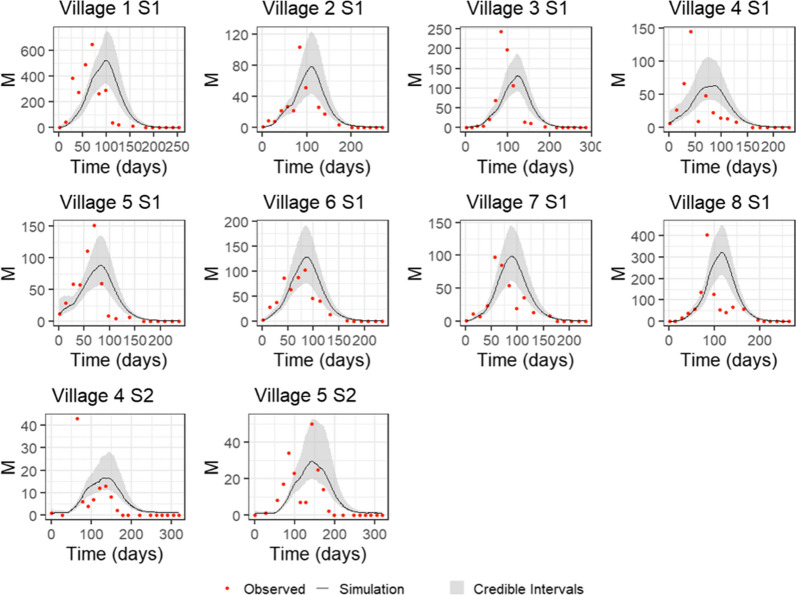
Model fits to Garki Project data for *non-clumped* egg-laying when fitting a power to density dependence with 95% credible intervals. Red points show counts of adult female mosquitoes (*M*) aggregated over individual villages for the first recorded rainy season in the data; for villages 4 and 5, a second rainy season denoted by S2 is also fitted to. Parameters for simulations were obtained from the median posteriors estimated by pMCMC fitting and 95% credible intervals from repeated samples of the joint posterior estimate

The pMCMC chains gave good convergence, and the model visually fitted the village-level time series well, both when using a linear (Eq. 2) and fitted power (Eq. 3) density dependence (see Fig. 2 and Additional file 1). Final parameter estimates were taken as the median of the posterior estimates with 95% credible intervals obtained by repeatedly sampling parameter sets from the joint posterior, Table 1. It was not possible to fit an exponential density dependence (Eq. 4), with the pMCMC algorithm unable to obtain reliable posterior estimates for any parameters, suggesting that exponential density dependence is highly unlikely. Deviance information criterion (DIC) values show that fitting a power provided the best model, 1104.149, compared to 1115.039 for linear density.

**Table 1:** Model parameters with priors, posterior estimates and 95% credible intervals for linear and fitted power density dependence, clumped and non-clumped egg-laying

Parameter	Description	Unit	Prior	Prior distribution	Posterior clumped	Posterior non-clumped
					Fitted power Linear	Fitted power Linear
$S$	Duration of gonotrophic cycle	Days	3	Fixed	–	–
$\beta$	Number of eggs laid per day per adult	Eggs day^−1^	1–35	Uniform	–	11.502 (2.544–26.585) 1.305 (1–2.372)
$n$	Clutch size	Eggs	1–35	Uniform	12.049 (2.945–28.051) 3.19 (1.563–5.925)	–
$d_{E}$	Development rate early larval instars	Days^−1^	0.150 (0.09–0.207)	Normal	0.15 (0.091–0.209) 0.147 (0.085–0.205)	0.149 (0.091–0.205) 0.140 (0.081–0.202)
$d_{L}$	Development rate late larval instars	Days^−1^	0.240 (0.037–0.428)	Normal	0.223 (0.102–0.373) 0.216 (0.100–0.374)	0.217 (0.100–0.391) 0.181 (0.100–0.343)
$d_{p}$	Development rate pupae	Days^−1^	1.00 (0.566–1.458)	Normal	0.884 (0.325–1.499) 0.855 (0.246–1.442)	0.826 (0.214–1.400) 0.639 (0.200–1.299)
$u_{E}^{0}$	Per capita mortality rate of early instars	Days^−1^	0.035 (0.022–0.047)	Normal	0.035 (0.022–0.048) 0.035 (0.022–0.048)	0.035 (0.022–0.048) 0.036 (0.022–0.048)
$u_{L}^{0}$	Per capita mortality rate of late instars	Days^−1^	0.035 (0.022–0.047)	Normal	0.036 (0.023–0.049) 0.035 (0.023–0.048)	0.036 (0.023–0.049) 0.035 (0.023–0.049)
$\mu_{P}$	Per capita mortality rate of pupae	Days^−1^	0.25 (0.184–0.318)	Normal	0.251 (0.184–0.317) 0.252 (0.184–0.319)	0.253 (0.184–0.317) 0.255 (0.188–0.321)
$\mu_{{\text{M}}}$	Per capita mortality rate of adults	Days^−1^	0.091 (0.0812–0.101)	Normal	0.090 (0.08–0.099) 0.089 (0.078–0.099)	0.089 (0.080—0.099) 0.089 (0.079–0.100)
$\tau$	Period of rainfall contributing to carrying capacity	Days	7.00 (2.000–12.000)	Normal	12 (8–16) 14 (8–19)	12 (8–15) 15 (11–19)
$\Omega$	Fitted power	–	–	–	0.322 (0.165–0.576) –	0.251 (0.171–0.352) –
$\gamma$	Effect of density dependence on late instars relative to early instars	–	13.06 (8.137–18.029)	Normal	13.317 (8.48–18.196) 13.016 (7.925–18.066)	13.441 (9.144–18.802) 13.027 (8.037–18.071)
$p$	Proportion of population sampled by trapping	–	–	–	0.029 (0.004–0.068) 0.024 (0.005–0.064)	0.03 (0.005–0.076) 0.026 (0.004–0.076)
$r$	Level of overdispersion	–	–	–	0.016 (0.002–0.04) 0.013 (0.002–0.036)	0.016 (0.002–0.044) 0.014 (0.002–0.043)
$I$	Immigration	Females per $\delta t$	1–8	Uniform	1.032 (0.213–3.897) 1.500 (0.264–4.569)	0.924 (0.161–3.156) 1.258 (0.224–4.478)
$\delta t$	Discrete time step	Days^−1^	0.25	–	–	–

Credible intervals were obtained by repeatedly sampling from the joint posterior distribution

### Egg clumping and clutch size estimates

**Fig. 3:**
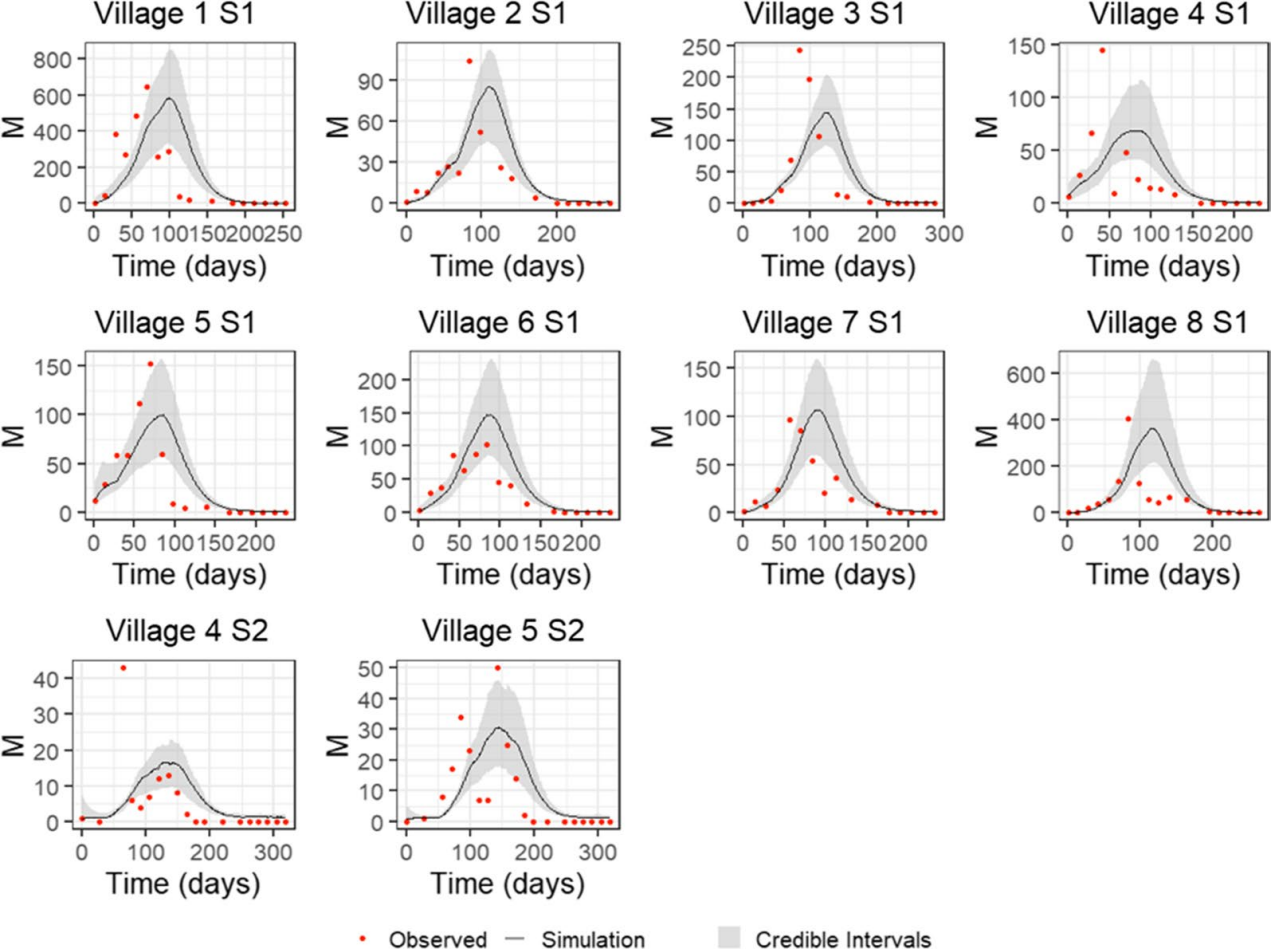
Model fits to Garki Project data for *clumped* egg-laying when fitting a power to density dependence with 95% credible intervals. Red points show counts of adult female mosquitoes (*M*) aggregated over individual villages for the first recorded rainy season in the data; for villages 4 and 5, a second rainy season denoted by S2 is also fitted to. Parameters for simulations were obtained from the median posteriors estimated by pMCMC fitting and 95% credible intervals from repeated samples of the joint posterior estimate

For both clumped and non-clumped egg-laying, when using a linear (Eq. 2) and fitted power density (Eq. 3) dependence, the model fitted to the data well. Again, however, it was not possible to fit an exponential density dependence (Eq. 4). DIC values show that fitting a power provided the best model, 1107.822, compared to 1122.032 for linear density. These values also show that non-clumped egg-laying provides a better fit to the data under both density dependence regimes, although this difference is marginal, particularly when fitting a power. Simulations of mosquito population dynamics using median posterior parameter estimates are shown in Figs. 2 and 3 for non-clumped and clumped egg-laying, respectively, of the fitted power density dependence (linear density dependence fits can be seen in Additional file 1).

Clutch size $n$ for fitted power (Eq. 3) and linear density dependence (Eq. 2) was estimated at 12.049 (95% CI 2.945–28.051) and 3.19 (95% CI 1.563–5.925), respectively. When considering non-clumped egg-laying, eggs laid per day $\beta$ was estimated as 11.502 (95% CI 2.544–26.585) for fitted power and 1.305 (95% CI 1.00–2.372) for linear. Note that, as previously described, for the continuous (non-clumped) egg-laying model variant, when estimating $\beta$, the gonotrophic cycle $S$ is not explicitly considered (unlike in the clumped egg-laying equation); therefore, a comparative value to $n$ (i.e. number of eggs laid in the same period) would be $\frac{{ \beta \left( {e^{{S\mu_{{\text{m}}} }} - 1} \right)}}{{\mu_{{\text{m}}} }}$, giving 39.566 (95% CI 8.751–91.449) for the fitted power and 4.489 (95% CI 3.439–8.157) for linear density dependence.

**Fig. 4:**
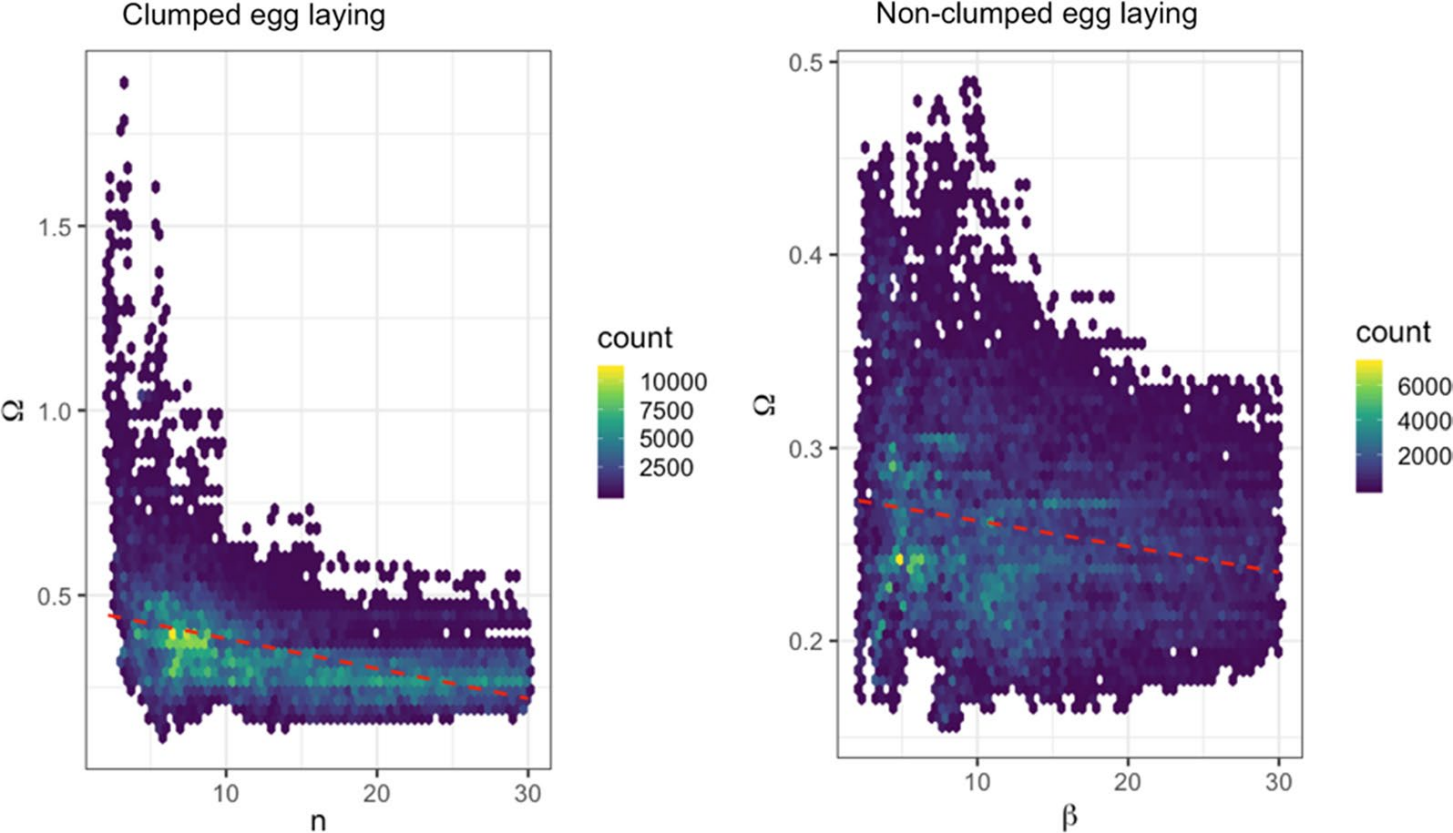
Cross-correlation plots between $n$ and $\Omega ,$ showing the corresponding two dimensions of parameter space explored by the pMCMC algorithm; the hexagon colour and count value represent the number of accepted parameter proposals

High levels of correlation between $\Omega$ and egg-laying also occurred during model fitting, where lower input of eggs into the system compensates for an increase in density-dependent mortality (Fig. 4). This is particularly pronounced under clumped egg-laying scenarios, showing that clutch size and the form of density dependence are intrinsically linked.

### Mosquito population growth rate

We found that simulated and raw analytical estimates of *R*_m_ were marginally lower for clumped egg-laying than continuous oviposition (Table 2), reflecting the finite lower bound on density-dependent mortality in the clumped model. However, for both clumped and non-clumped egg-laying, *R*_m_ was significantly lower when considering a linear density dependence over a fitted power.

**Table 2:** *R*_m_ estimates with 95% credible intervals for clumped and non-clumped egg-laying under linear and fitted power density dependence

	Clumped		Non-clumped	
	Linear	Fitted power	Linear	Fitted power
Simulated	3.099 (2.027–4.408)	13.532 (3.775–26.543)	3.407 (2.506–4.883)	31.958 (10.891–71.342)
Analytical	4.007 (2.6–6.148)	16.314 (4.588–34.256)	5.449 (3.778–7.721)	45.321 (13.708–90.157)

*R*_m_ is calculated in two ways, numerically by simulating the model for a single female mosquito until its death in a system empty of other adults and recording the number of female offspring surviving to adulthood, and analytically as described in the methods. For the *R*_m_ estimate simulations, the median parameters from the pMCMC posterior estimates were used. To estimate 95% credible intervals, 500 parameter sets were randomly taken from the pMCMC results and the *R*_m_ simulation run 50 times with these values and a mean taken. The credible intervals were then estimated from the resulting 500 mean values

### Effect on gene drive

**Fig. 5:**
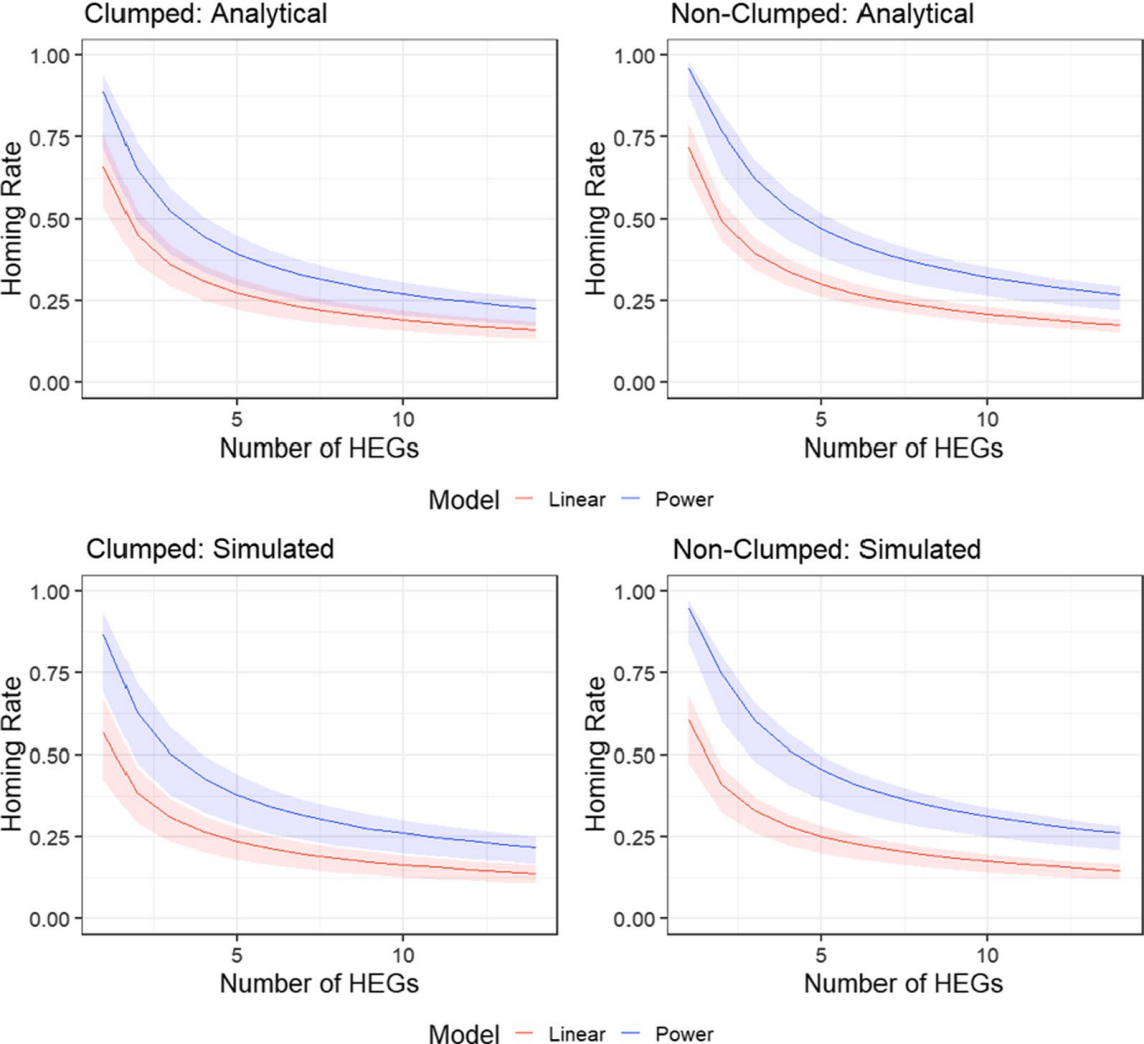
Repeat of model by Deredec et al. [20] using parameters estimated by our analysis, *R*_m_ was derived both analytically and numerically for all density and egg-laying scenarios. The model estimates the number of HEGs needed in relation to their homing rate (a measure of efficacy) for successful elimination of a mosquito population. The bold centre line is the parameter estimates from the median of the posterior; the shaded bands represent the 95% credible intervals

Both clumped egg-laying and fitting a power to the density dependence affected the required number and homing rate of HEGs for successful population elimination (Fig. 5). As expected, this is in line with the changes Deredec et al. [20] found when they modified values for *R*_m_. Changes in density dependence had the greatest effect, whilst clumped egg-laying showed a relatively minor effect that cannot be concluded to be significant due to the overlap of credible intervals.

## Discussion

Here we have quantified the effects of differing density dependence regimes and mosquitoes laying their eggs in a clumped, non-homogeneous fashion. From this we can see how both egg-laying and density dependence have a clear impact on estimates of *R*_m_ and subsequent vector control efficacy.

Much recent development work on genetically modified mosquitoes has aimed at increasing homing and transmission rates of constructs [44–47]. In this context, our analysis suggests that the challenges to elimination posed by high values of *R*_m_ could vary substantially, especially if density dependence and egg-laying regimes are not universal. The consequences of this finding could have a significant impact on both the efficacy required from gene drive constructs and the release strategies necessary for successful deployment both positive and negative. Numerical and analytical estimates of *R*_m_ were significantly lower under a linear density dependence and clumped egg-laying scenario than under continuous egg-laying and a fitted power density dependence (Table 2). This level of reduction in *R*_m_ is substantial when considering predictions of the required homing rate and number of HEGs from our runs of the model by Deredec et al. [20].

Whilst we have looked only at HEG efficacy in this study, the variation in *R*_m_ will also be further compounded by wild-type resistance. For example, in work by Beaghton et al*.* [45] looking specifically at mutation rates in driving-Y interventions, *R*_m_ was key to the probability of mutations arising. This occurs because, with increasing *R*_m_, the time to elimination is increased, and thus the probability that resistant mutations arise before population elimination is achieved. In addition, the probability of a resistant mutation becoming established also increases, as at a higher *R*_m_ the probability of stochastic loss decreases.

The fitting of a value for $\Omega$ < 1 for both clumped and non-clumped egg-laying scenarios suggests a negative, non-linear density effect, i.e. as the larval population increases towards the environmental carrying capacity, the rise in severity of density effects decreases. It could be suggested from these results that whilst the overall death rate in the population is increasing, at the individual scale the mortality rate is decreased. This could be explained by there being a protective effect or efficiency gain in foraging/energy expenditure within larger groups of larvae, as often seen in other organisms [48, 49]. Additionally, the effects of density dependence may be mitigated to an extent by deceased or smaller larvae acting as an alternate food source.

The difference in *R*_m_ estimates between density dependence regimes is quite stark; however, it is difficult to identify the most probable functional form, particularly as DIC values are relatively close. In the wild, there is likely variation between sites and locations which can impact on larval development and adult fitness. These may be dependent on multiple factors, e.g. highly localised microclimates or differences in faunal communities. What this study perhaps more clearly shows is the importance of understanding this component of life history and the need for empirical data to help predict density dependence effects. This is something which was also highlighted in Khamis et al. [29], who similarly identified how density dependence could have a significant impact on vector control and cost-effectiveness. Additionally, our *R*_m_ estimates of non-clumped laying are lower than those from the comparable deterministic model by White et al. [28], likely a result of the more realistic, stochastic nature of our model and the fitting techniques used.

Estimated values for *n* and $\beta$ in our study are relatively low, and lower than reported clutch sizes of *Anopheles*. However, the class *E* in our model is not a value for eggs, but early instar larvae, and thus will be lower than actual egg numbers when egg survival/hatching is considered. In addition, as the model does not include space or attempt to model individual breeding sites, the representation of density is not precise.

Generally, our model fitted to the data well, despite over- or underestimating some of the more extreme data points, particularly the higher peaks. However, it is important to consider how much overdispersion is inherent in such population counts. Further, as our model does not simulate individual breeding sites, there is likely variation between and within villages we do not capture. For example, we assume a single mosquito species and do not consider the interactions and competition with other species in larval pools. Whilst the environmental niches of mosquitoes both within and outside the *Anopheles gambiae* complex vary (some species such as *An. arabiensis* prefer larger or more permanent pools, whilst *An*. *gambiae* s.s. prefer smaller temporary sites [50]), there is still the possibility of interspecies competition for resources, and transient environmental conditions may lead to temporary niche overlap. As rainfall leads to higher carrying capacity, it also affects the type of habitat available, which may vary between villages/sites, and this could lead to a shift in species-specific density dependence effects. Lastly, in focusing solely on density dependence in larval mortality, our model does not account for density or other detrimental effects in the adult population, such as the size and availability of blood meals and infection status [51, 52], or the adult fitness costs resulting from density-dependent impeded larval development [53–55].

## Conclusions

In this study, we have identified how general assumptions over egg-laying and the functional form of density dependence in mathematical models can have a significant impact on the estimates of life-history parameters. These in turn fundamentally affect the likely impact of vector control interventions such as gene drive. Our results suggest that these aspects of mosquito ecology should not be ignored in future studies, and understanding of local population dynamics are vital in our predictions of vector control efficacy. To build on this work, future data collection should consider detailed temporally regular, and preferably spatially stratified, population counts of adults and larvae in highly seasonal settings to better characterise these effects. Further, it is important we understand how these effects may change under differing regimes of mosquito and aquatic diversity, predator abundance and infection status, necessitating the need for comprehensive longitudinal studies conducted in realistic environments.

## Supplementary Information

**Additional file 1.** Detailed model fitting methods and additional results.

## Data Availability

Data is publicly available at http://garkiproject.nd.edu.

## Code availability

Code is available at: https://github.com/aaronm70/mosModel.
