# Supplementary material for: Fine-scale estimation of key life-history parameters of malaria vectors: implications for next-generation vector control technologies
Source: Parasit Vectors. 2021 Jun 8;14:311. doi: 10.1186/s13071-021-04789-0 (PMC8188720; doi:10.1186/s13071-021-04789-0)
Supplement: Supplementary file 1 — Additional file 1. Detailed model fitting methods and additional results. [file 13071_2021_4789_MOESM1_ESM.pdf]

Additional file 1

Table of Contents

Deriving initial conditions 2

Particle filter algorithm and likelihood methods. 4

Linear density dependence model fits 6

Priors and posterior estimates from pMCMC for clumped and non-clumped egg laying 8

References 12

Deriving initial conditions

To minimise computational effort, we estimated a single value from which initial conditions for all mosquito life stages (*E*, *L*, *P* and *M*) can be derived. This means during pMCMC only one value (z) needs to be proposed for, rather than four. To calculate values for *E*, *L*, *P* and *M* from z, the model must first be transcribed into the following set of differential equations:

$$\frac{dE}{dt} = \beta M - d_E E - \mu_E^0 \left(\frac{E(t)+L(t)}{K(t)}\right)^{\Omega} E$$

$$\frac{dL}{dt} = d_E E - d_L L - \mu_L^0 \left(\gamma \frac{E(t)+L(t)}{K(t)}\right)^{\Omega} L \qquad (1)$$

$$\frac{dP}{dt} = d_L L - d_P P - u_P P$$

$$\frac{dM}{dt} = \frac{1}{2} d_P P - u_M M$$

These equations can then be set to zero and solved to equilibrium to get the following set of equations:

$$a = \frac{\beta\, d_p\, d_L}{2\mu_M(\mu_p + d_P)}$$

$$b = \frac{{\mu_p}_E^0}{{\mu_p}_L^0 \gamma}\left(d_L + {\mu_p}_L^0\right) - d_E - {\mu_p}_E^0$$

$$c = \frac{{\mu_p}_E^0\, d_E}{{\mu_p}_L^0 \gamma}$$

$$x = \frac{b + \sqrt{b^2 - 4ac}}{2a} \qquad (2)$$

$$z = L = \left(\frac{d_E - d_L - u_L^0}{{\mu_p}_L^0 \gamma}\right)\frac{1}{\Omega}\ \left(\frac{K}{x+1}\right)$$

$$E = \frac{L}{x}$$

$$P = \frac{d_L L}{\mu_p + d_P}$$

$$M = \frac{d_p P}{2\mu_M}$$

Using these, we can estimate a single parameter value for z which for these equations is equal to L, and from this calculate values for E, P and M. As the estimation of z encompasses Ω the equations can be used both for linear, where $\Omega = 1$, and fitted power density dependencies.

For an exponential density dependence, we can again solve the differential equations and fit a single value z, from which it is possible to derive E, L P and M using the following set of equations:

$$a = \frac{\frac{1}{2} dLdP}{\mu M(dP + \mu P)}$$

$$E = \frac{\beta a z}{dE + \beta a + \mu_E^0 \, e^{\frac{z}{K}}}$$

$$L = \frac{dEz}{dE + dL + \mu_E^0 \, e^{\gamma \frac{z}{K}}} \tag{3}$$

$$P = 2\mu M \frac{M}{dP}$$

$$M = \frac{\frac{1}{2} dLdPL}{\mu M(dP + \mu P)}$$

Particle filter algorithm and likelihood methods.

Particle filters are an algorithm which apply a form of importance re-sampling to generate an approximate sample from and make inferences about an unobserved markov process (1, 2). For example, the marginal likelihood $p(y|\theta)$ for the data y with the parameter vector θ is considered the joint posterior probability $p(y|x,\theta) \times p(x|\theta)$, where x is the trajectory of the simulation. Using monte-carlo approximation for $p(y|\theta)$, a particle filter with J particles which have the possible trajectories of x_j the marginal likelihood can be considered as $p(y|\theta) \approx \sum_J p(y|x_j,\theta) \times p(x_j|\theta)$

To run the particle filter algorithm requires the following steps:

1. Initialise the particles with equal weights.

$$x_j \sim p(x_j|\theta) \tag{4}$$

$$w_j = \frac{1}{J}$$

2. For each particle j at time t, simulate the initial conditions at the first observed data point.

$$x_{jt} \sim p(x_{jt}|x_{j,}\theta) \tag{5}$$

3. Calculate a probability weighting for each particle based on the results of the simulation and the observed data.

$$w_{jt} = p(y_t|x_j,\theta) \tag{6}$$

Probability weightings were derived from a beta-binomial function to account for overdispersion, where B is the beta distribution which takes two shape parameters (a,β), p is the fraction of the population being fitted to and r is the level of overdispersion in the data giving:

$$p(y_t|x_{jt},\theta) = \binom{x_{jt}}{y_t}\left(\frac{B\left(x_{jt}+p\left(\frac{1}{r}-1\right),y_t+(1-p)\left(\frac{1}{r}-1\right)\right)}{B\left(p\left(\frac{1}{r}-1\right),(1-p)\left(\frac{1}{r}-1\right)\right)}\right) \tag{7}$$

4. Take the average probability weighting for all particles, which can be considered the marginal likelihood for this data point.

$$p(y_t|\theta) = \frac{1}{J}\sum\nolimits_J w_{jt} \quad (8)$$

5. Normalise the weightings $\frac{w_{jt}}{\sum_J w_{jt}}$, resample with replacement each particle based on their weighting and simulate forward to the next observed data point.

$$x_{jt+1} \sim p\left(x_{jt+1}\middle|x_t, \theta\right) \quad (9)$$

Repeat steps 3 to 5 for all observed data, an estimate for the total likelihood value can be considered the product of the marginal likelihood at each step.

$$\mathcal{L}(\theta|y_{1:t}) = \prod_{n=1}^{t} p(y_n|x_n, \theta) \quad (10)$$

Linear density dependence model fits

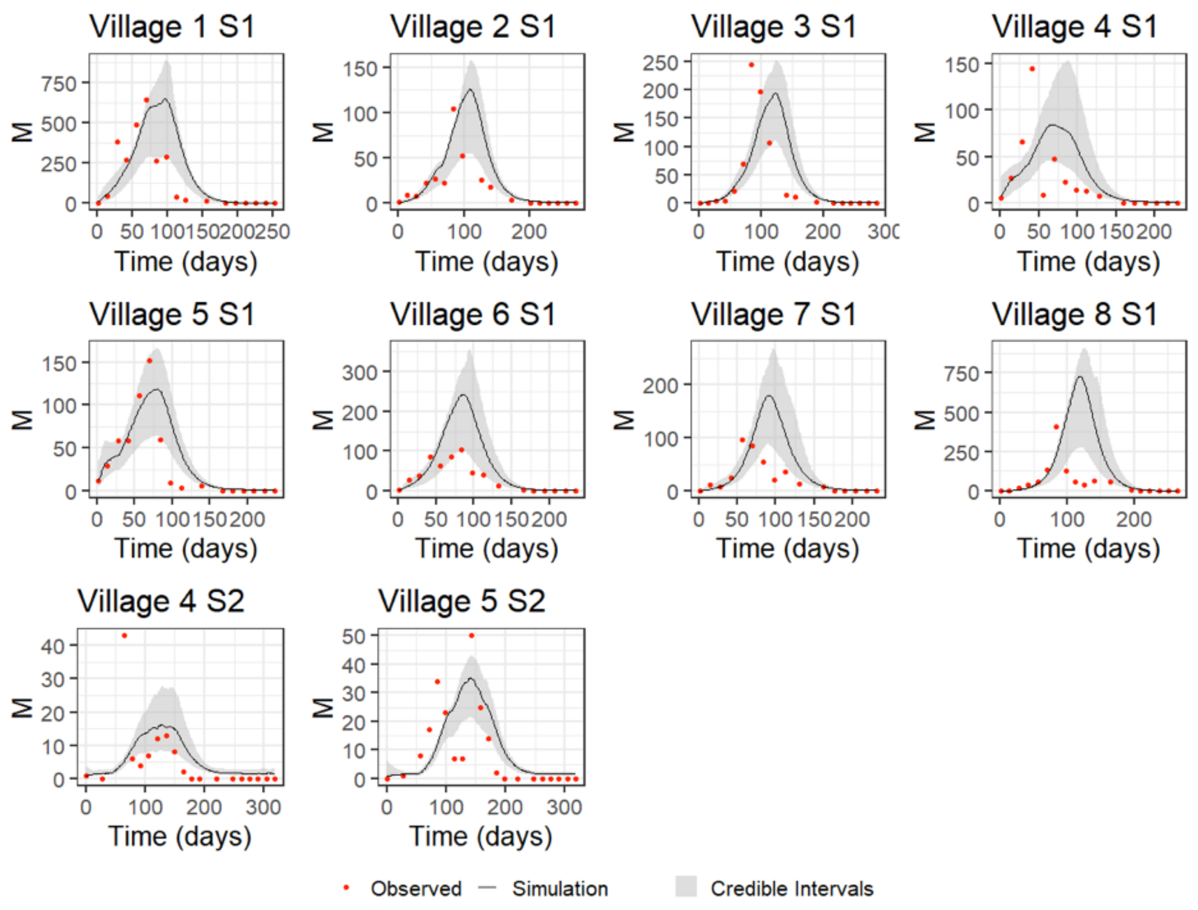

Figure S1 Model fits to Garki Project data for **clumped** egg laying with linear density dependence. Black points show counts of adult female mosquitoes (M) aggregated over individual villages for the first recorded rainy season in the data, for villages 4 and 5 a second rainy season denoted by S2 is also fitted to. Parameters for simulations were obtained from the median posteriors estimated by pMCMC fitting.

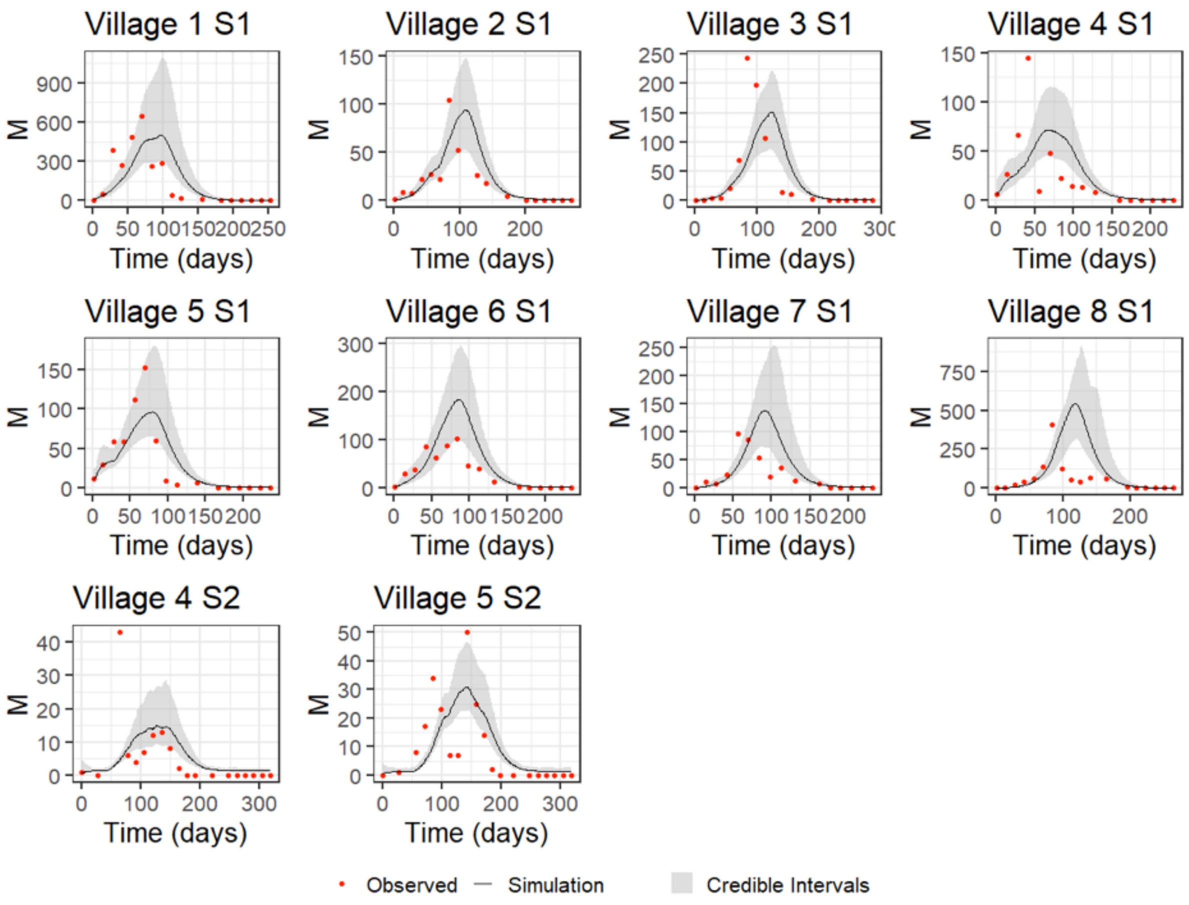

Figure S2 Model fits to Garki Project data for **non-clumped** egg laying with linear density dependence. Black points show counts of adult female mosquitoes (M) aggregated over individual villages for the first recorded rainy season in the data, for villages 4 and 5 a second rainy season denoted by S2 is also fitted to. Parameters for simulations were obtained from the median posteriors estimated by pMCMC fitting.

Priors and posterior estimates from pMCMC for clumped and non-clumped egg laying

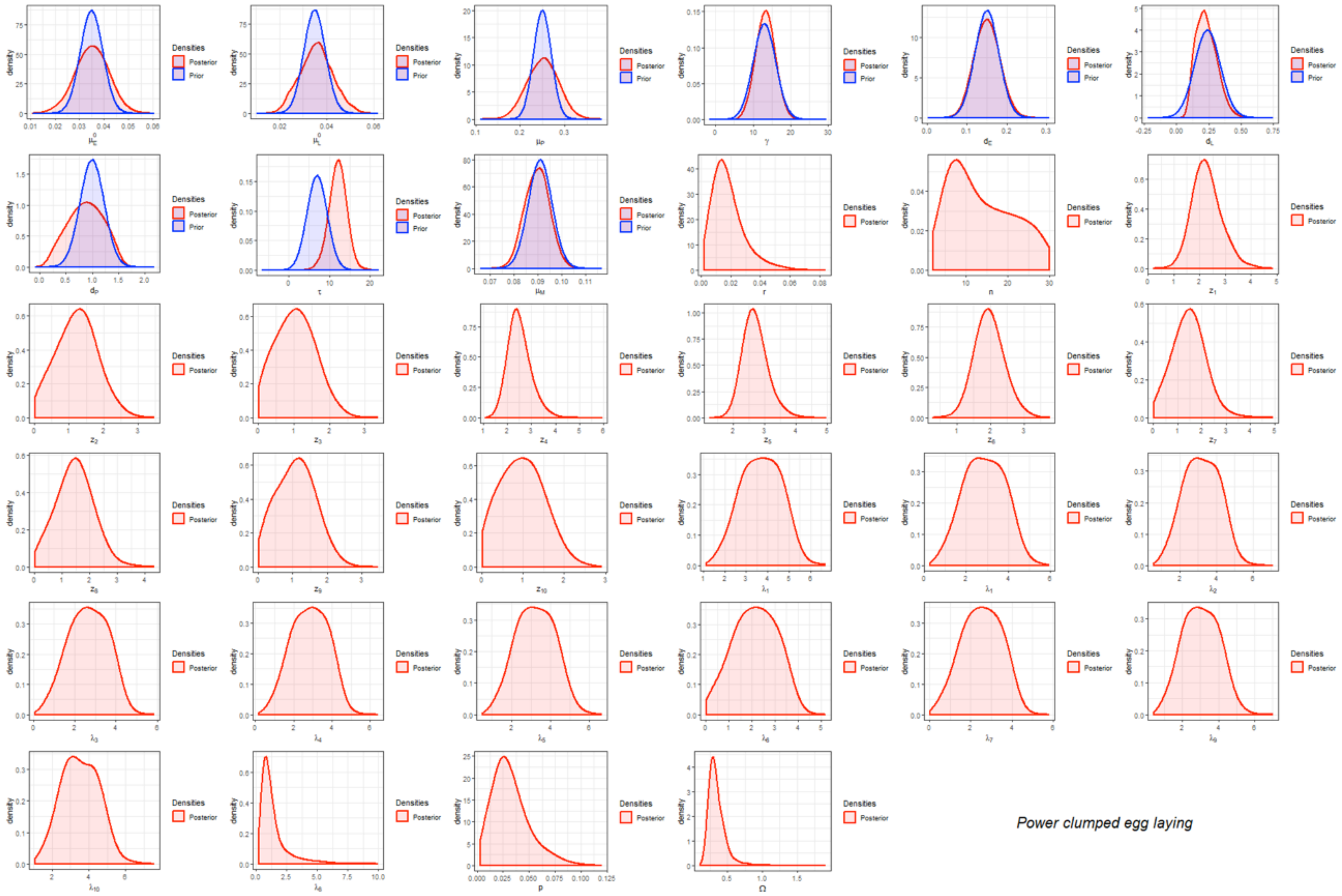

Power clumped egg laying

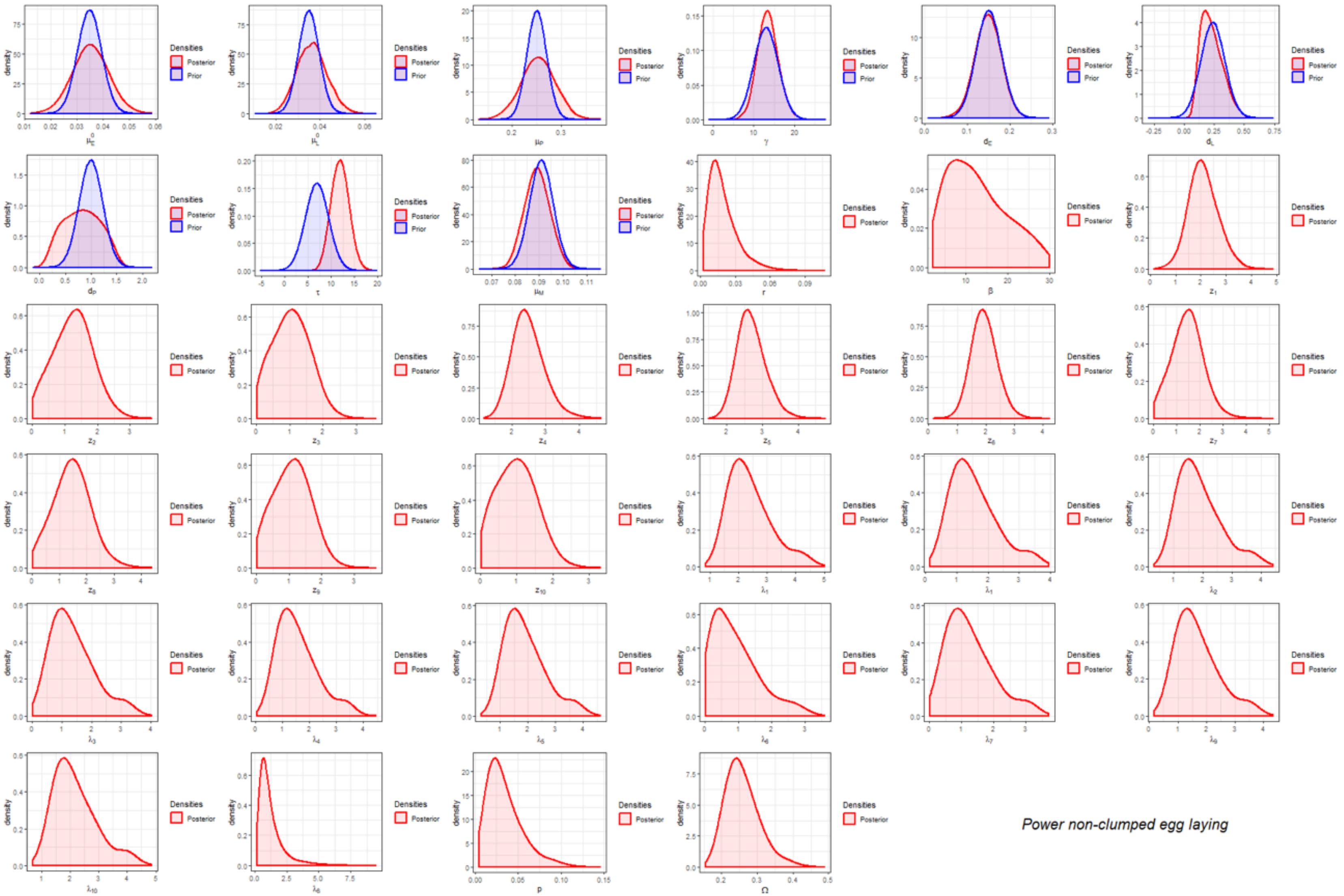

Power non-clumped egg laying

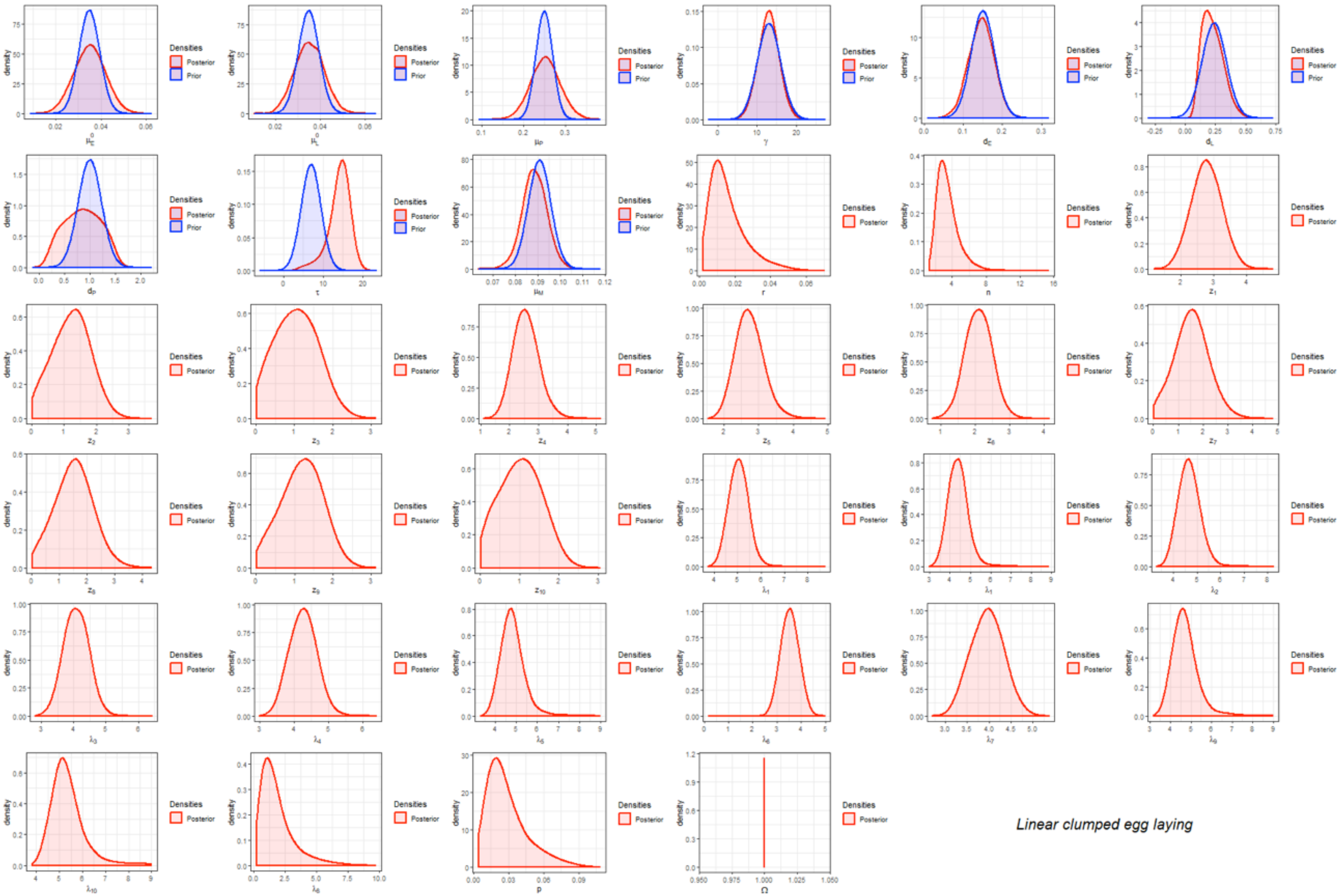

Linear clumped egg laying

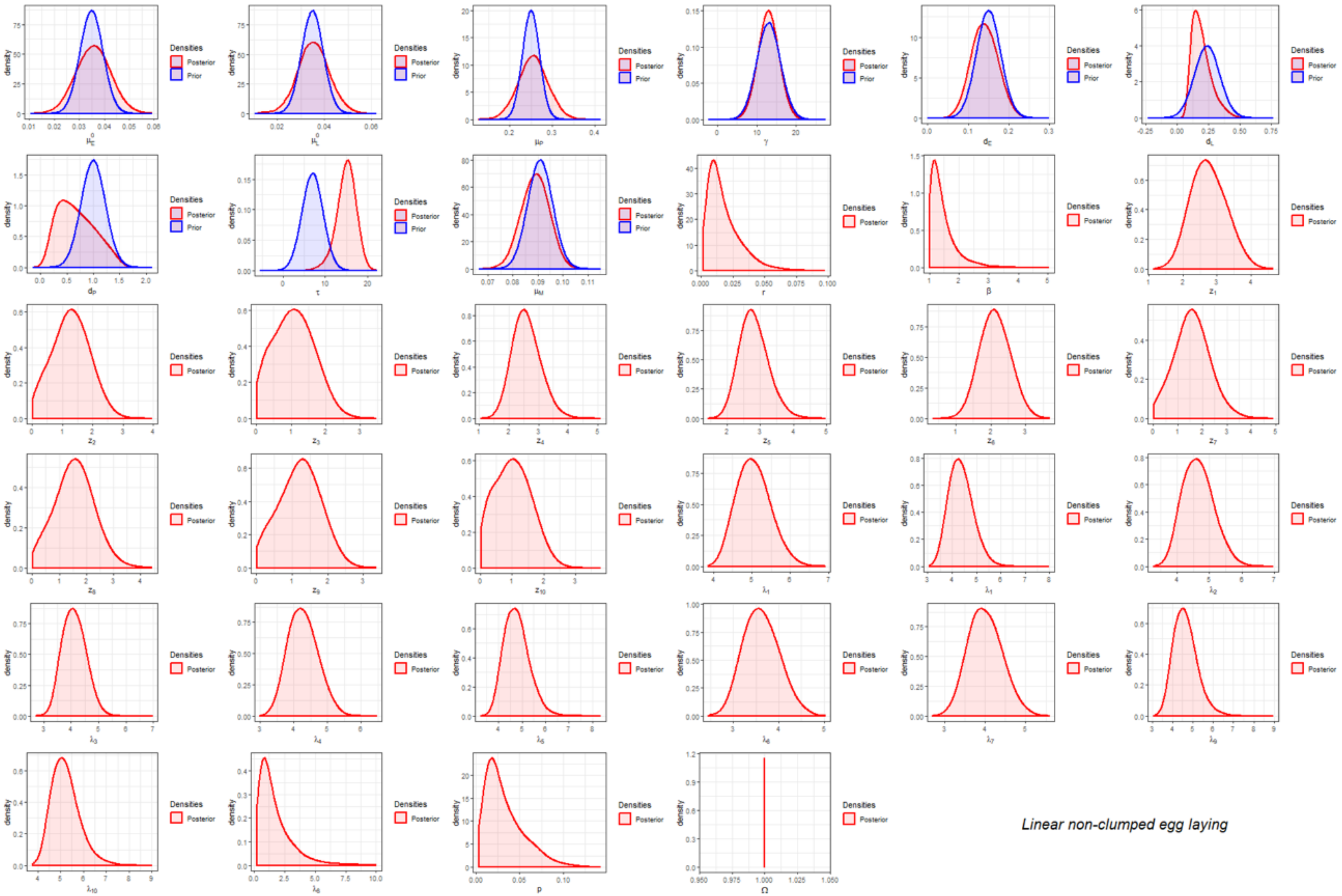

Linear non-clumped egg laying

References

1. A. Doucet, S. Godsill, C. Andrieu, On sequential Monte Carlo sampling methods for Bayesian filtering. *Statistics and computing* **10**, 197-208 (2000).
2. N. J. Gordon, D. J. Salmond, A. F. M. Smith (Novel approach to nonlinear/non-Gaussian Bayesian state estimation. (IET), pp 107-113.
